# Integrating transcriptome and microRNA analysis identifies genes and microRNAs for AHO-induced systemic acquired resistance in *N. tabacum*

**DOI:** 10.1038/s41598-017-12249-y

**Published:** 2017-10-02

**Authors:** Yongdui Chen, Jiahong Dong, Jeffrey L. Bennetzen, Micai Zhong, Jun Yang, Jie Zhang, Shunlin Li, Xiaojiang Hao, Zhongkai Zhang, Xuewen Wang

**Affiliations:** 1 Biotechnology and Germplasm Resources Institute, Yunnan Academy of Agricultural Sciences; Yunnan Provincial Key Laboratory of Agricultural Biotechnology; Key Lab of Southwestern Crop Gene Resource and Germplasm Innovation, Ministry of Agriculture, Kunming, 650223 P. R. China; 2 Germplasm Bank of Wild Species, Kunming Institute of Botany, Chinese Academy of Sciences, 132 Lanhei Road, Kunming, 650201 P. R. China; 3 China Tobacco Gene Research Center, Zhengzhou Tobacco Research Institute of CNTC, Zhengzhou, 450001 P. R. China; 4 State Key Laboratory of Phytochemistry and Plant Resources in West China, Kunming Institute of Botany, Chinese Academy of Sciences, Kunming, 650201 P. R. China; 5 Department of Genetics, University of Georgia, Athens, USA

## Abstract

3-Acetonyl-3-hydroxyoxindole (AHO) induces systemic acquired resistance (SAR) in *Nicotiana*. However, the underlying molecular mechanism is not well understood. To understand the molecular regulation during SAR induction, we examined mRNA levels, microRNA (miRNA) expression, and their regulatory mechanisms in control and AHO-treated tobacco leaves. Using RNA-seq analysis, we identified 1,445 significantly differentially expressed genes (DEGs) at least 2 folds with AHO treatment. The DEGs significantly enriched in six metabolism pathways including phenylpropanoid biosynthesis, sesquiterpenoid and triterpenoid biosynthesis for protective cuticle and wax. Key DEGs including *PAL*s and *PR-10* in salicylic acid pathway involved in SAR were significantly regulated. In addition, we identified 403 miRNAs belonging to 200 miRNA families by miRNA sequencing. In total, AHO treatment led to 17 up- and 6 down-regulated at least 2 folds (Wald test, P < 0.05) miRNAs (DEMs), respectively. Targeting analysis implicated four DEMs regulating three DEGs involved in disease resistance, including miR156, miR172f, miR172g, miR408a, *SPL6* and *AP2*. We concluded that both mRNA and miRNA regulation enhances AHO-induced SAR. These data regarding DEGs, miRNAs, and their regulatory mechanisms provide molecular evidence for the mechanisms involved in tobacco SAR, which are likely to be present in other plants.

## Introduction

Systemic acquired resistance (SAR) is an induced defense response that confers long-lasting protection against a broad spectrum of pathogen infections^1,2^. Localized treatment of plants with activators, compounds controlling disease without directly affecting the pathogen, results in the development of enhanced resistance against pathogens in the entire plant. Resistance induced by such activators is generally characterized by restriction of pathogen growth and decreased disease severity^3,4^. Only a few compounds have been found to induce SAR in plants^2,5^. One example, Acibenzolar-S-methyl (ASM) stimulates the production of plant defense-related compounds^6,7^. However, no chemical treatment that can completely inhibit pathogen infection is available.

Natural products from plants have been proven to be a good resource in antimicrobial research, because plants have already evolved multiple mechanisms to selectively suppress pathogens by production of secondary metabolites with antimicrobial activities. 3-Acetonyl-3-hydroxyoxindole (AHO), a derivative of isatin, has been isolated from extracts of *Strobilanthes cusia* ^8^. AHO induces resistance in tobacco plants against infection with *tobacco mosaic virus* (TMV), the fungal pathogen *Erysiphe cichoracearum* (powdery mildew) and tospoviruses^9^, possibly through the salicylic acid pathway-mediated SAR^8^. However, the molecular mechanism of how AHO induces SAR has not been fully elucidated.

Plants have inducible defense mechanisms to protect from pathogen attacks. The inducible physiological defense of plants often includes rapid and localized cell death known as the hypersensitive response (HR), which is mediated either by gene interaction between a plant resistance (*R*) gene and a pathogen avirulence (*Avr*) gene or by the binding of a nonrace-specific elicitor, such as elicitin, to a putative receptor^10^. In addition, redox regulators, the mediator complex, transcription factors, endoplasmic reticulum-resident proteins, and DNA repair proteins also play critical roles in SAR^4^. To date, only a few genes have been shown to be involved in plant SAR, including gene expression that leads to increased salicylic acid (SA)^1,11–13^. A transcriptional co-activator encoded by the gene *NPR1* plays a role in the SA signaling during basal resistance against pathogens^14^. Pathogenesis-related (PR) proteins are also in the SA signaling pathway, and some are also induced by various abiotic stresses^15^, and AHO-induced SAR increases *PR* expression in tobacco^8^. PRs are known to function in cell wall rigidification, signal transduction and antimicrobial activity^16^. Phenylalanine ammonia lyase (PAL) is a key enzyme for SA synthesis^17^. AHO-induced plant disease resistance is accompanied by increased PAL activity and SA accumulation^8^. Jasmonic acid (JA) is known to trigger plant immunity^18^. The increased JA level during pathogen perception promotes the *CORONATINE INSENSITIVE1* (*COI1*) expression, which represses the expression of *Jasmonate ZIM domain* (*JAZ*) and promotes degradation of JAZ. Then JAZ’s repression on several transcription factors is released, which increases the expression of defense genes^18^. SAR and plant disease resistance are complicated biological processes involving numerous genes and proteins. These components deserve further investigation.

MicroRNAs (miRNAs) are non-coding RNAs that are 20–24 nucleotides in length^19–21^. miRNA functions as guide RNAs to direct the repression of their mRNA targets at post-transcriptional level. Some of these target genes include those involved in hormone signaling, responses to biotic stresses and response to abiotic stresses^22–27^. Studies have suggested that miRNAs function in the plant defense reaction. The precursor miRNA ptc-MIR408 suppresses the expression of *PALs*. MiRNAs regulate several plant hormone signaling pathways of SA, abscisic acid (ABA) and jasmonic acid (JA)^18,27^. Down-regulation of JA biosynthesis by miR319 promotes SA-mediated resistance responses^27,28^. MiRNAs regulate the expression of protein phosphatase 2C (PP2C) and laccase^29,30^. MiR398 targets at enzyme superoxide dismutase (SOD) which is involved in the synthesis of antibacterial substances^31,32^. Because of the conserved regulatory functions of miRNA, miRNA-guided post-transcriptional regulation is expected to be involved in the response to infections in most plants. Therefore, the identification of stress-associated miRNAs should help understand the regulation at miRNA level during AHO-induced SAR in tobacco.

RNA-seq technology has been widely used to discover key genes and quantifying gene expression. This include pathogen resistance genes in *Brassica napus* ^15^, key genes for nitrogen utilization in the tea plant^33^, and genes for sugar metabolism in date palm^34^. Because miRNAs recognize their mRNA targets via base paring, the mRNA targets can be predicted using bioinformatics software. Hence, integrated mRNA and miRNA analysis has proven to be an effective method for exploring the molecular network at transcriptional and post-transcriptional levels in response to biotic and abiotic stresses in plants^35–39^.

To better understand the molecular network in AHO-induced SAR, we applied both RNA-seq for transcriptome analysis and miRNA-seq for miRNA analysis in our study. Our results suggested that the AHO induces SAR in tobacco, and we identified differentially expressed genes and differentially expressed miRNAs (DEMs) in SAR. We predicted the mRNA targets of DEM, and predicted the regulatory interactions between specific DEM and specific DEGs. Our results help to explain the molecular mechanisms of SAR at the transcriptional and post-transcriptional levels. Because aspects of SAR are evolutionarily conserved across species, our results should provide a useful resource for SAR investigations in other plants.

## Results

### Inhibitory effect of AHO on *tomato spotted wilt virus* (TSWV) disease

**Figure 1:**
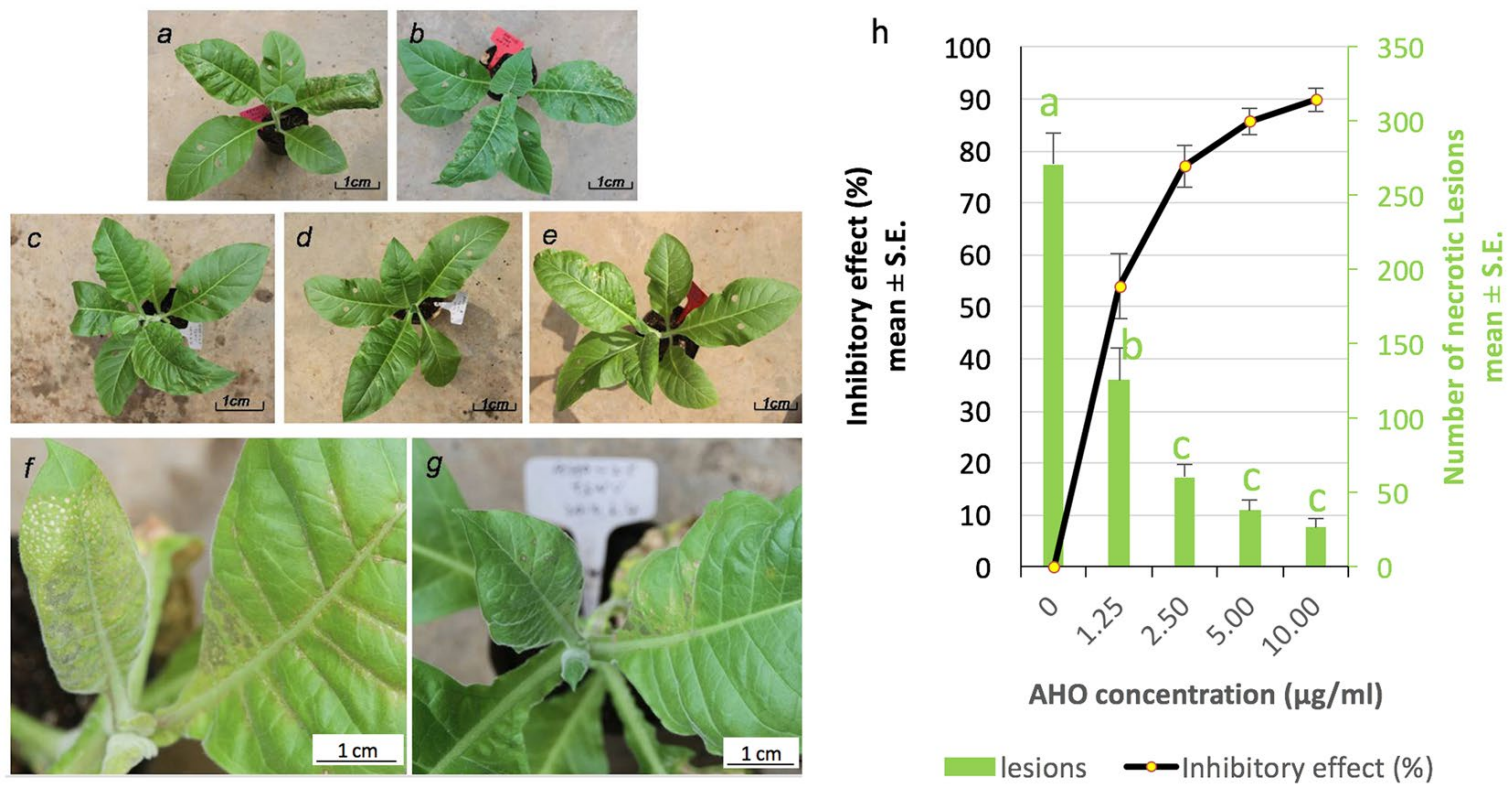
Tobacco plants were sprayed with equal amounts of DMSO solution with different concentrations of AHO. Tomato spotted wilt virus was then inoculated mechanically on two middle leaves of each of three to five plants 24 hours after spraying. Photos were obtained after five days. (**a**–**e**) represents the tobacco plant treated with 0, 1.25, 2.5, 5.0 and 10.0 µg/ml AHO, respectively. (**f**,**g**) shows detailed infection in leaves of control and plants treated with 10 µg/ml AHO. Image h represents the inhibitory effect was calculated as the percent of (the total necrotic lesions in each control plant – that in each AHO-treated plant)/the total number of necrotic lesions in each control plant × 100%. One-way ANOVA was used for statistical analysis between treatment and control. Letter a, b and c in image (**h**) represent significance level at 0.01 compared with each other.

TSWV induces necrosis in tobacco plants^7^. To evaluate the inhibitory effect of AHO on TSWV, we inoculated *N. tabacum* K326, treated with different concentrations of AHO, with TSWV and recorded the disease symptoms. Our results showed that typical necrotic lesion symptoms caused by TSWV appeared in leaves after five days of inoculation in control plants (Fig. 1). After 15 days, more necrosis was observed in veins, old leaves, and the newly developed leaves in the control plants. In contrast, significantly less necrosis (ANOVA test P < 0.01) appeared on the AHO-treated leaves, especially in the treatment groups that received a high concentration of AHO treatment groups (Fig. 1f–g). The 10 µg/ml AHO treatment had an inhibitory rate of 89.9% on TSWV symptoms (Fig. 1, Table S1). Taken together, we concluded that AHO-treated plants had a significantly less TSWV infection than the control, thus suggesting that AHO induced SAR.

### RNA-seq assembly and gene annotation for *N. tabacum* K326

**Figure 2:**
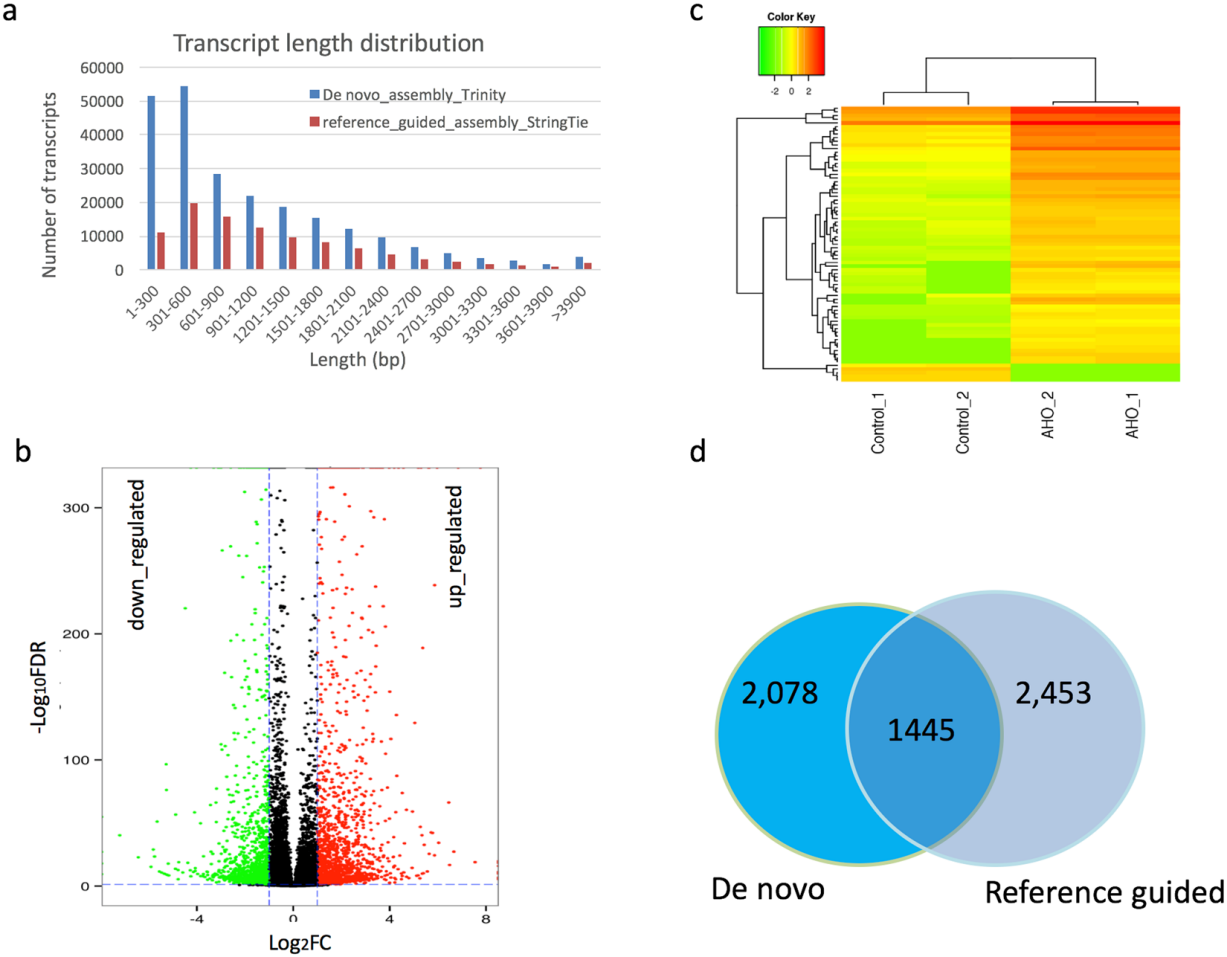
Transcripts length and AHO induced differential gene expression in *N. tabacum* leaves. Image (**a**) shows the comparison of length distribution of transcripts which were de novo assembled and referenced guided assembly. Image (**b**) presents the differentially expressed genes (FDR < 0.05 and > = 2-fold change). Image (**c**) presents genes expression with at least 4-fold changes in gene expression with AHO treatment compared with control. Color represents the level of expression in FPKM after log2 transformation. _1 and _2 represent the two experimental repeats. Image (**d**) represents the shared DEGs from both assembly analyses.

To understand gene expression during AHO-induced SAR, we examined the transcriptomes in leaves of *N. tabacum* treated with 0 or 10 µg/ml AHO, by using Illumina HiSeq 2500 RNA-seq technology. A total of 83,753,496 clean 100-bp paired-end reads with quality Q30 > 91% were generated. To get the transcriptome accurately, we used two transcript assembling methods, the reference sequence guided and de novo assembly, to construct the transcript. The very recent updated *N. tabacum* K326 assembly^40^ was used as the reference to guide transcript assembly, and this method generated 99,547 transcripts from 66,700 super gene loci by using software Hisat and StringTie according to previously described protocols^41^. The gene number obtained here is close to the estimate number 69,500^40^. Of these gene loci, 37,759 (57%) were identical to existing gene annotation^40^. On average, each gene produced 1.4 transcripts. 85,461 (26%) and 35,675 (16%) novel exons and intron were found compared with existing genome annotation^40^, respectively. While de novo assembling with the Trinity software (version Trinityrnaseq_r20160317) according to the published protocol^42^ generated 236,647 transcripts belonging to 95,422 predicted unigenes, of which 26,972 were longer than 600 bp. The N50 length of transcripts from both methods were very similar, 1,775 bp in de novo assembly and 1,791 bp in reference guided assembly (Table S2). Comparison of transcription length revealed that *de novo* assembly generated more short transcripts than reference guided assembly (Fig. 2a), especially in <600 bp transcripts (Table S2). For both methods, more than 74% of the reads can be pair-mapped into transcripts or genome reference concordantly. The RNA-seq reads and assembly information have been deposited at NCBI and are publicly available under Bioproject number PRJNA342398 and accession number GFCB01000000, respectively. All unigenes from de novo transcripts were then used to search, via BLAST (threshold E-value 10-5), against each of the seven databases of NR, Swissprot, GO, COG, KOG, Pfam, and KEGG for annotation. The reference annotation of *N. tabacum* K326 at NCBI was chosen as the priority if available. By this analysis, a final total 37.8% of the unigenes were assigned a functional annotation from the best BLAST hit from at least one database, and the annotated transcript are mainly longer (>300 bp) transcripts (Table S3).

### Differential transcript levels in response to AHO

To reveal the DEGs in SAR induced by AHO, we measured transcript levels in fragments per kilobase per million mapped reads (FPKM) from RNA-seq data. Then, we compared the differentially expressed transcripts. After AHO treatment, 3,523 *de novo* unigenes in leaves were identified as DEGs with at least 2-fold change (false discovery rate <0.05) by using the previously described protocol^42^. Of these, 1,971 and 1,552 genes were up- and down-regulated, respectively (Fig. 2b). Further analysis revealed that 75 genes displayed at least 4-fold changes. Of these genes, 70 genes exhibited increased transcript level, whereas five transcripts exhibited decreased level (Fig. 2c). While from the reference guided assembly, we found 3,898 DEGs with at least 2-fold change (Wald test, P < 0.05) by using the tool DESeq2^43^. Of these, 2,150 and 1,748 genes were up- and down-regulated, respectively. Of the two sets of assembled DEGs, 1,445 DEGs were shared though they were constructed from different methods (Fig. 2d).

### Functional analysis of differentially expressed genes

**Figure 3:**
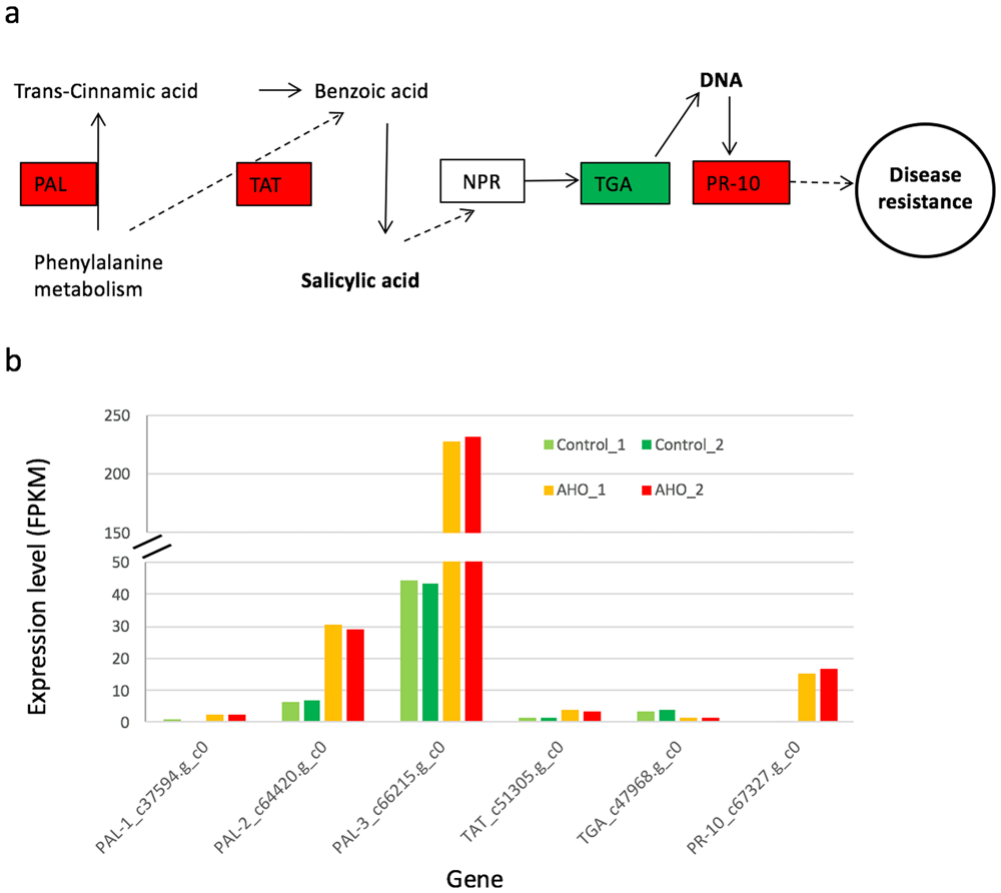
Regulation of genes in salicylic acid pathway. The putative pathway (image **a**) was modified from KEGG pathway 04075 (version 9/6/16). Red and green represents the differentially up-regulated and down-regulated gene expression (image **b**) with AHO treatment in *N. tabacum* K326, respectively. Boxed are proteins or enzymes encoded by corresponding genes with same name.

To better understand the function of the DEGs detected between the AHO-treated and control samples, we mapped the shared 1445 DEGs into the KEGG pathway database and identified enriched pathways by using the tool KAAS^44^. The DEGs were significantly enriched (hypergeometric test, P < 0.05) in six KEGG pathways in plants by using R package GOseq^45^. The enriched pathways included phenylpropanoid biosynthesis (KEGG pathway ID ko00940), sesquiterpenoid and triterpenoid biosynthesis for protective cuticle and wax^46^ (KEGG pathway ID ko00909), biosynthesis of secondary metabolites (KEGG pathway ID ko01110), and photosynthesis - antenna proteins (KEGG pathway ID ko00196), etc (Table 1). As the phenylalanine involved in phenylpropanoid metabolism and is the substrate molecule for SA biosynthesis^12,47^, we mapped the DEGs to the SA pathway (KEGG map ID 04075)^48^ in *Arabidopsis* and found the homologous gene in tobacco (Fig. 3a). We identified three homologs (*c37594.g_c0*, *c64420.g_c0* and *c66215.g_c0*) of *PAL* genes in SA biosynthesis, and the *PAL*s expression was up-regulated (Fig. 3b). We also identified an up-regulated homolog (*c51305.g_c0*) of tyrosine aminotransferase (TAT) gene known for SA synthesis, and an up-regulated pathogen-related gene *PR-10* homolog (*c67327.g_c0*) in the SA pathway, consistent with that reported previously^49^. The homolog of transcription factor TGA known to be involved in the SA pathway^50^ was down-regulated. In addition to SA, we assessed the transcript level for homologous genes involved in JA pathway and found only the *JAZ* gene (*c64585.g_c0*) was up-regulated while level of other genes like *COI1* in this pathway were not changed.

**Table 1:** Enriched differentially expressed genes in pathways at KEGG database.

Pathway ID	over_represented P value	under_represented P value	Number of DEGs	Number of total genes	Pathway Name
ko04111	0.002	1	22	26	Cell cycle
ko00196	0.003	0.999	28	39	Photosynthesis - antenna proteins
ko04113	0.009	0.999	14	16	Meiosis
ko00940	0.992	0.02	51	114	Phenylpropanoid biosynthesis
ko00909	0.992	0.02	6	29	Sesquiterpenoid and triterpenoid biosynthesis
ko01110	0.999	0.002	90	206	Biosynthesis of secondary metabolites

Enrichment analysis was conducted using length-bias corrected statistical method from R package GOseq^45^.

### miRNA identification and its abundance measurement

**Figure 4:**
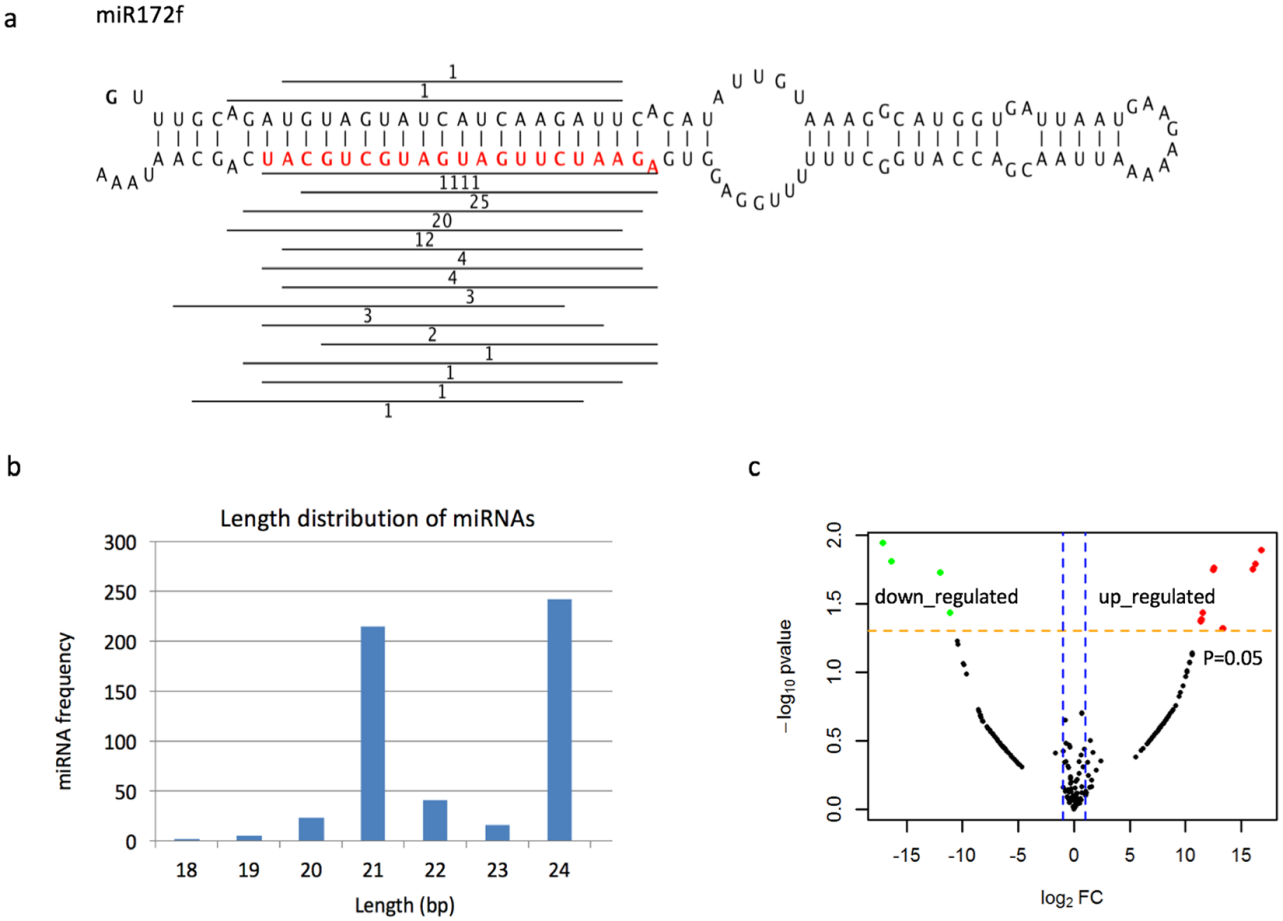
miRNA identification, distribution and differential regulation. Image (**a**) presents the hairpin structure of miR172f as an example of identification of miRNAs from *N. tabacum* k326. The mature miRNA sequence is presented in red. The lines above or under the mature miRNA represent the matching reads in miRNA sequencing data, and the numbers represent the count of aligned reads. Image (**b**) presents the length distribution of miRNAs. Image (**c**) presents the distribution of differentially expressed miRNAs.

To reveal the role of miRNAs in the SAR induced by AHO, we sequenced all miRNA candidates with the Illumina GAIIx (Illumina, USA) platform and obtained 15.4 million and 15.8 million single-end reads from the control and AHO treatment, respectively. To increase the accuracy of miRNA identification, we combined all read data and the publicly available genome assembly of *N. tabacum* K326 as a reference to mine the miRNA candidates with miRPlant software (version 4)^51^. After removing rRNAs, tRNAs and snoRNAs from among the candidates by using Blastn tool, we identified 403 miRNAs (Fig. 4a) belonging to 200 miRNA families (Table S4). These identified miRNAs were supported by robust genomic sequence and mapped miRNA reads. Therefore, the miRNA set identified was more accurate than that previously reported from genomic DNA in *N. tabacum* ^52,53^. After comparing the results, we identified 36 conserved miRNA families that were present in miRbase (version 21), whereas 164 miRNA families from our dataset were absent, thus suggesting that most of the miRNAs identified from *N. tabacum* K326 in this study were novel (Table S4). The 21-bp and 24-bp miRNAs were most abundant (Fig. 4b). Multiple members of the same miRNA family were discovered in the study. miR5303, novel107, miR156, miR166 and miR396 had 32, 24, 23, 14 and 10 members in each family, respectively (Table S4). Multiple members of miR156, miR166 and miR396 have also been reported in a previous study^54^. All other novel miRNA families had fewer than 5 members. We found that abundance of 115 miRNAs were at least 2-fold different in AHO treated versus untreated tobacco leaf controls. Although some previous studies only used 2 folds as a criterium to determine DEMs, we here used probability to increase the statistical power of the DEM identification with the tool DEseq2^43^. We identified that 23 miRNAs exhibited a significant (Wald test, P < 0.05) abundance difference of at least 2 folds (Fig. 4c). Of these miRNAs, 17 and 6 DEMs members were significantly up-regulated and down-regulated, respectively (Fig. 4c, Table S4).

### miRNA regulation of target gene expression

To explore the biological significance of DEMs, we analyzed the putative miRNA-target mRNA using the plant-specific prediction tool psRNATarget with strict parameter settings^42^. We predicted that 23 miRNA members targeted 16 DEGs in *de novo* assembly with AHO treatment (expectation cut off < = 2, Table S5). These miRNAs belonged into eight conserved and eight novel miRNA families (Table S5). Among these miRNAs, four members belonged DEMs and were predicted to regulate three DEGs (Table 2), thus indicating likely interactions of miRNA and mRNA in SAR (Table S5). We then compared with these target genes’ abundance in reference guided assembly, and revealed that all three genes were DEGs too with the same expression change patterns (Table 2).

**Table 2:** Differentially expressed miRNAs and their differentially expressed target genes.

Differential expressed miRNA					Differentially expressed gene				
miRNA	Log_2_(FC)	P value	Expression	Inhibition	Predicted target gene	FDR^#^ P value*	Log_2_(FC)	Expression	Predicted gene function
miR156v	12.5	0.02	up	Cleavage	c32948.g_c0^#^ N.11366*	5E-04^#^ 0.004*	−1.8^#^ −1.4*	down	squamosa promoter-binding-like protein 6, transcription factor SPL6
miR172f	11.4	0.04	up	Cleavage	c56968.g_c0^#^ N.9090*	3E-12^#^ 0.002*	2.4^#^ 2.3*	up	AP2-like ethylene-responsive transcription factor TOE3 (AP2), binds to the GCC-box pathogenesis-related promoter element
miR172g	11.4	0.04	up	Cleavage				up	
miR408a	−11.1	0.03	down	Translation	c23376.g_c0^#^ N.38165	6E-112^#^ 4E-06*	2.1^#^ 3.7*	up	a blue protein with copper ion binding function for electron transport

Change was supported by transcript levels in both de novo assembling assay (#) and reference guided assembly assay (*). FDR represents false discovery rate. The predicted target gene marked by # or * are the same gene from corresponding assembly method.

**Figure 5:**
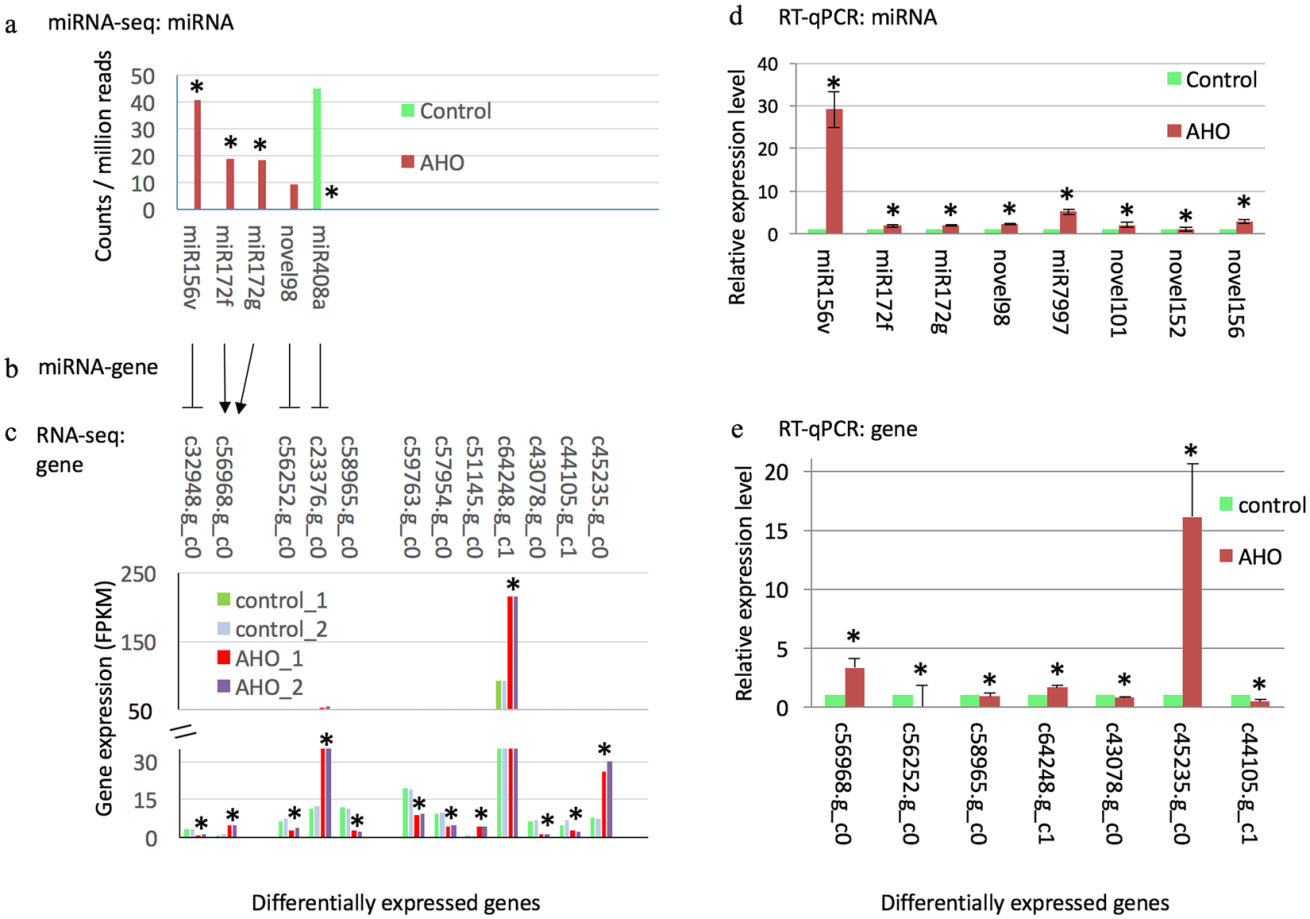
Expression of miRNAs and their target transcripts. Image (**a**) shows the DEMs abundance change in RNA-seq analysis with AHO treatment. Image (**b**) represents the potential regulation between miRNAs and their target genes. The T shape end and arrow represent the opposite or same regulation trend between miRNA and mRNA. Image (**c**) shows the DEGs level in RNA-seq analysis. Image (**d**) and (**e**) shows the relative expression levels of miRNA and mRNA transcript assessed by qRT-PCR, respectively. The relative level of qRT-PCR calculated by the delta-delta-CT method. Data is presented as mean ± standard error.

Of the three targeted DEGs, two genes were found to encode transcription factors, while the other gene encoded a protein with copper ion binding function (Table 2). We observed two patterns of miRNA-targeted gene expression. Pattern A. One miRNA targeted only one target DEG (Table 2). We predicted that increased miR156v levels are at least partly responsible for the down-regulation of DEG *c32948.g_c0* during SAR (Fig. 5a,c). Gene *c32948.g_c0* is predicted to encode transcription factor SPL6, a required key transcriptional regulator in resistance to TMV in tobacco and against *Pseudomonas syringae* in *Arabidopsis* ^55^. The miR156v down-regulation on *SPL6* in SAR is consistent with previous finding in pathogen defense in *Arabidopsis* ^56^ (Table 2, Fig. 5a,b,c). MiRNA 408a was down-regulated while its predicted targeted DEG *c23376.g_c0* exhibited up-regulated transcript levels after AHO treatment (Table 2, Fig. 5a,b,c). Gene *c23376.g_c0* encodes a predicted protein with copper ion binding activity in electron transport, which has been proposed to be involved in redox reactions occurring during primary defense responses in plants^57^. Pattern B. Several miRNAs appear to target one DEG. Three members in the miR172 family have the structural properties that would allow them to regulate DEG *c56968.g_c0*, encoding an AP2-like ethylene-responsive transcription factor (AP2) which has been predicted to bind to the GCC-box pathogenesis-related promoter element and to respond to environmental stimuli^58,59^. Only abundance of miRNA172g and miRNA172f were significantly increased (>2 folds and Wald test, P < 0.05) after AHO treatment. The relationship is that the expression of DEG *c56968.g_c0* was increased although miRNA172g and miRNA172f were up-regulated (Table 2, Fig. 5a,b,c), suggesting that greater transcription of DEG *c56968.g_c0* after AHO treatment overcomes the higher levels of post-transcriptional inhibition of miRNAs for this locus.

### RT-qPCR validation of levels of mRNAs and miRNAs

We used real-time quantitative PCR (RT-qPCR) to validate the expression level of selected interesting DEGs and DEMs, including specific DEMs and its targeted DEGs (Table S6). We tested eight interesting DEMs including miR156v, miR172f, miR172g, novel98 etc. and seven interesting DEGs (*c56968.g_c0, c58965.g_c0, c64248.g_c1, c43078.g_c0, c44105.g_c1, c45235.g_c0* and *c56252.g_c0*). The test included miRNA-target pairs including DEMs of miR172f and miR172g, and their target DEG *c56968.g_c0*. We also tested the miRNA novel98 with 10-fold change but statistically not significant (Wald test, P = 0.8) and its predicted target DEG *c56252.g_c0*. The same patterns of change in RNA levels were observed in qRT-PCR as that in the RNA-seq analyses (Fig. 5d and e). Interestingly, we found that the change of novel98 level in qRT-PCR was significantly, suggesting that it should pay caution to draw conclusion on expression level with a high fold change but no statistical significance (P > 0.05) in sequencing result.

## Discussion

Some chemical compounds can induce SAR in plants^1,2,5^, and therefore can have wide applications in agriculture. However, the molecular mechanisms underlying the induction of SAR by natural products extracted from plants have rarely been investigated. A previous study has revealed that the compound AHO induces SAR together with changes in PR-1 gene expression, salicylic acid content, and PAL activity^8^. Our study further investigates molecular mechanisms of AHO-induced SAR in *N. tabacum*. Here, we focused on the genome-wide regulation of gene expression, miRNA expression, and predicted interaction between miRNAs and mRNA-encoding genes. We confirmed that AHO induced SAR in *N. tabacum* through TSWV inhibition experiments, and obtained expressed gene profiles from these *N. tabacum* leaf tissues. The reference guided assembly detected a slightly higher transcripts number (99,549) than the estimated ~93,000 in the tobacco K326 draft genome^60^. In contrast, the *de novo* assembly generated 2.6 times transcripts belonging to 95,422 unigenes, which is a standard observation for de novo RNA-seq analysis because several unigenes can often be derived from a single protein-coding gene because of incompleteness RNA-seq data contig generation or transcript clustering. This can be inferred from more short transcripts in de novo constructed transcripts than the reference guided transcripts. The RNA levels of 75 genes was found to differ by more than 4 folds. A 4-fold change in RNA level suggests a dramatic increase or decrease in expression level caused by regulating gene expression up or down, but it can also be an outcome of changes in the stability of individual mRNAs.

In our analysis, more genes were found to increase their transcript levels than to decrease with AHO treatment. This finding suggests that AHO-driven SAR activated more gene expression, thereby increasing acquired resistance in plants. These up-regulated genes may play important roles in SAR and are worthy of further investigation. Our results revealed that the AHO-treatment and resultant SAR generated a complicated set of responses enriched by DEGs in six metabolism pathways, including phenylpropanoid biosynthesis (KEGG pathway ID ko00940), sesquiterpenoid and triterpenoid biosynthesis for protective cuticle and wax^46^ (KEGG pathway ID ko00909), and biosynthesis of secondary metabolites (KEGG pathway ID ko01110). Some DEGS were interesting because of they have been known as involving in SAR. A DEG example associated with unigenes ID *c67327.g_c0* was up-regulated in tobacco leaves treated with AHO. This gene is annotated as encoding STH-2-like PR protein (also known as PR-10a^49^), which plays a crucial role against viral infection in hot peppers^49^. PR-10 gene expression is regulated by plant hormones and defense-related signaling molecules, including JA and SA^61^. We have previously observed increased SA in AHO-induced SAR^8^, which may have up-regulated PR-10 expression in this study. Phenylalanine is the source for SA biosynthesis through the PAL enzymatic pathway^12,47^. Here, we also identified that *PALs*, *TAT* genes involved in SA synthesis were up-regulated. This suggests these genes in SA pathway play roles in AHO-induced SAR, similar to their roles in plant response to pathogen.

The small non-coding RNAs called miRNAs regulate numerous aspects of post-transcriptional gene expressions and have potential applications in crop improvement^62^. However, the roles of miRNAs in SAR in plants are not fully characterized. Although studies have reported the discovery of miRNAs in *N. tabacum* ^52,53^, the miRNAs were found to be at a low confidence by automated evaluation in the miRBase database (version 21). The reason for this low confidence was the previous lack of *Nicotiana* genomic sequences for use in miRNA prediction. In this study, we used the publicly available *N. tabacum* K326 draft genome sequence as the reference to validate the mined miRNAs from K326. We mined the miRNAs and then aligned the miRNAs to genomic sequence to confirm their origin and to improve accuracy. We identified 403 miRNA members and categorized them into 200 families. The majority of miRNA are 21 or 24 nucleotides in size, as also seen in previous report^16^. Most of the miRNAs were novel, and some miRNAs, such as miR172, had multiple members. These data indicated that the miRNAs discovered here are more accurate and abundant than those previously reported. We further calculated miRNA abundance and identified 17 and 6 miRNAs that were up- and down-regulated significantly (Wald test, P < 0.05) in AHO-induced SAR, respectively. The DEMs identified here have higher confidence with the statistical power than most of other DEMs associated published articles which used only 2 folds of abundance change.

Plant miRNAs have conserved regulatory function. We explored the molecular network between miRNA and its target mRNA in SAR by predicting the miRNA and its targeted DEG. MiRNAs are known to functionally down-regulate target gene expression^22–27^, as reflected in pattern A, whereby one DEM is predicted to repress one DEG. We found predicted interaction in this category for miR156v and miR408a (Fig. 5a,b and c). We also predicted another pattern, pattern B, wherein multiple DEMs regulated one DEG, named *c56968g_c0*. The observed DEG level in pattern B may reflect the combined effects of multiple regulations. We did not identify miRNA targeting of reported genes^8^ that encode enzymes in the SA biosynthesis pathway or PR proteins.

We identified DEMs targeting DEGs that function in disease resistance, as supported by findings in other plants. We predicted two transcription factors SPL and AP2 are AHO-affected at the miRNA level, in agreement with that transcription factor mRNAs are the preferred targets of miRNAs^10^. In this study, the up-regulated miR156v is predicted to target the *c32948.g_c0* gene, which encodes the transcription factor SPL6. Numerous SPL genes are post-transcriptionally regulated by miR156^63,64^. SPL is potentially involved in divergent signal transduction pathways mediated by auxin, gibberellin, ethylene and other plant hormones^65^. Hence, our study confirmed that miR156v plays a role in plant disease resistance potentially through down-regulating SPL6 expression. We predicted that miR172f and miR172g targeted *AP2* (unigene ID *c56968.g_c0*). This regulation has been reported in plants^66^ and is involved in nodule formation by rhizobium infection in soybean^57^. Multiple miR172 members regulate *AP2*, and we found a net increase in miR172 levels that co-occurred with increased *AP2* level. This finding indicated that miR172 does not greatly suppress the net level of the *AP2* transcript. A similar phenomenon has been reported in which induced miR172 is associated with high levels of *AP2* transcript but no AP2 protein in *Arabidopsis* ^67^. Early studies have confirmed that miR172 regulates expression of the target gene *AP2* by cleavage of mRNA or translational inhibition^68^. Considering these results together, we hypothesize that miRNA172 may regulate *AP2* at the level of translational repression rather than mRNA cleavage in AHO-induced SAR. As a key transcription factor, it is expected that *AP2* will be regulated by many factors, including those that affect RNA level. Studies have revealed that plantacyanin-like (basic blue) protein is the target of miR408^66,69^. Here, we predicted that decreased miR408a targeted basic blue protein-like (DEG *c23376.g_c0*) which exhibited an up-regulation in mRNA level. The regulated protein may play a role in plant resistance through modulating the activities of oxido-reductase in the electron transport chain (Table 2). Future efforts are needed to undertake the extensive and challenging molecular genetics are needed to experimentally validate of the interplay of miRNA and mRNA target of miRNA156, 172 and miR408a.

The discussed DEGs and DEMs may be key players in AHO-induced SAR. However, it is also possible that the additional DEGs or DEMs may account for some of the AHO-induced SAR. In this regard, we identified several interesting DEGs in the AHO-treated tissues. For instance, the decreased expression of hexose carrier protein HEX6-like (DEG ID *c58965.g_c0*) (Table S5) could diminish the transport of hexose in AHO-treated leaves. The susceptibility of plants to disease is dependent on the sugar content in the leaf^70^, and sugars play an important role in the induction of defense responses^71^. A prerequisite for defense gene expression is a certain level of hexoses delivered by via a transporter^72,73^. As a consequence of perturbation of sugar metabolism, defense-related genes are activated, and SA levels are elevated, and SAR is consequently induced^72^. We also found that DEG *c51145.g_c0* encodes P2C protein (Table S5). P2C plays a crucial role in the ABA signaling pathway, as shown by studies in *Arabidopsis* and *P. patens* ^59,74^. The increased expression of *P5C* (DEG *c64248.g_c1*) in our study is also consistent with that reported in *N. benthamiana* during pathogen infection^75^. We found that *P5C* was up-regulated by ~ 3 folds (Fig. 5c).

## Conclusions

In summary, this is an initial report of gene regulations of AHO-induced SAR in the model plant *N. tabacum* K326. We generated transcriptome and miRNA sequence data for control and AHO-treated tobacco leaves and performed comprehensive analysis of the AHO-induced mRNAs and miRNAs. The results revealed DEGs potentially involved in AHO-induced SAR, including genes related to responses to plant hormone signal transduction, phenylalanine biosynthesis, pathogenesis-related protein biosynthesis and starch and sucrose metabolism. In addition, our analysis also identified miRNAs that may play important roles in AHO-induced SAR. Furthermore, potential regulatory interactions between specific miRNAs and their target transcripts were revealed. These findings provide valuable information regarding the roles of mRNA and miRNA at transcriptional and post-transcriptional levels in AHO-treated plant *N. tabacum*. It will be vital to see how similar the patterns of differentially expressed genes, miRNAs and their regulatory will be associated with SAR in other crops.

## Materials and Methods

### Plant materials and treatment

In an insect-free greenhouse, tobacco seeds of *Nicotiana tabacum* cv K326 were sown in seed pans containing a mixture of 50% (w/w) peat culture substrate, 40% (w⁄w) humus and 10% (w⁄w) perlite. Thirty to thirty-five days after seeding, seedlings were transplanted into pots (one plant per pot). Tobacco plants were cultivated to the 5~6-leaf stage before use in the experiments. Briefly, 0.1 g AHO was dissolved in 1 ml dimethyl sulfoxide (DMSO) and then diluted with distilled H_2_O to 1.25, 2.5, 5, or 10 μg/ml. DMSO without AHO was used as a control. Each tobacco plant was sprayed with 5 ml AHO solution or control solution. Three to five plants were used in each treatment. Two experimental repetitions were undertaken.

### Inhibitory effect of AHO on *tomato spotted wilt virus* symptomology

*Tomato spotted wilt virus* was mechanically inoculated on the two middle leaves of each plant 24 hours after spraying. Three-five plants were used for each treatment. These plants were cultivated in the greenhouse for 15 days. The first time of necrotic lesions appeared on the leaves was recorded. The number of necrotic lesions was counted, and the inhibition percentage was calculated by using the following formula (the total number of necrotic lesions on each control plant-the total number of necrotic lesions on each AHO-treated plant)/the total number of necrotic lesions on each control plant × 100%.

### RNA extraction and sequencing

For mRNA and miRNA analysis, all leaves were collected from each of three to five tobacco plants with 10 μg/ml AHO treatment or control at 6 h and were immediately frozen in liquid nitrogen. The leaf samples of each treatment were stored at −80 °C until use. Leaf samples were pooled for the same treatment and ground into powder in liquid nitrogen with a mortar and pestle. Total RNA was extracted from 80–120 mg powder by using TRIzol Reagent (Ambion) (Invitrogen Life Technologies, Shanghai, China, cat# 15596-026). The quality and quantity of total RNA were characterized on a 1% agarose gel and examined with a NanoDrop 2000c spectrophotometer (NanoDrop Technologies, Wilmington, DE, USA). The RNA integrity number (RIN) was assessed by using an Agilent 2100 Bioanalyzer (Santa Clara, CA, USA). The RIN of mRNA samples was greater than 8.0, and mRNA was used for subsequent Illumina library preparation. mRNA was enriched from 15 μg total RNA by using an NEBNext poly(A) mRNA Magnetic Isolation Module (NEB, cat# E7490L) and AMPure XP Beads (Beckman Coulter, Inc., cat# A63881). mRNA was cleaved into short fragments by using buffer and then indexed by nucleotide barcode. Then, the sequencing library was prepared with an NEBNext mRNA Library Prep Master Mix Set for Illumina (NEB, cat# E6110L) and NEBNext Multiplex Oligos for Illumina (NEB, cat# E7500). The library was then quantified by qRT-PCR using a Quantification Kit-Illumina GA Universal (Kapa, cat# KK4824). Paired-end 100-bp sequencing was performed for the qualified library on a HiSeq 2500 machine. Two duplicated experiments were conducted.

### Transcript assembly, annotation and expression analysis

**Figure 6:**
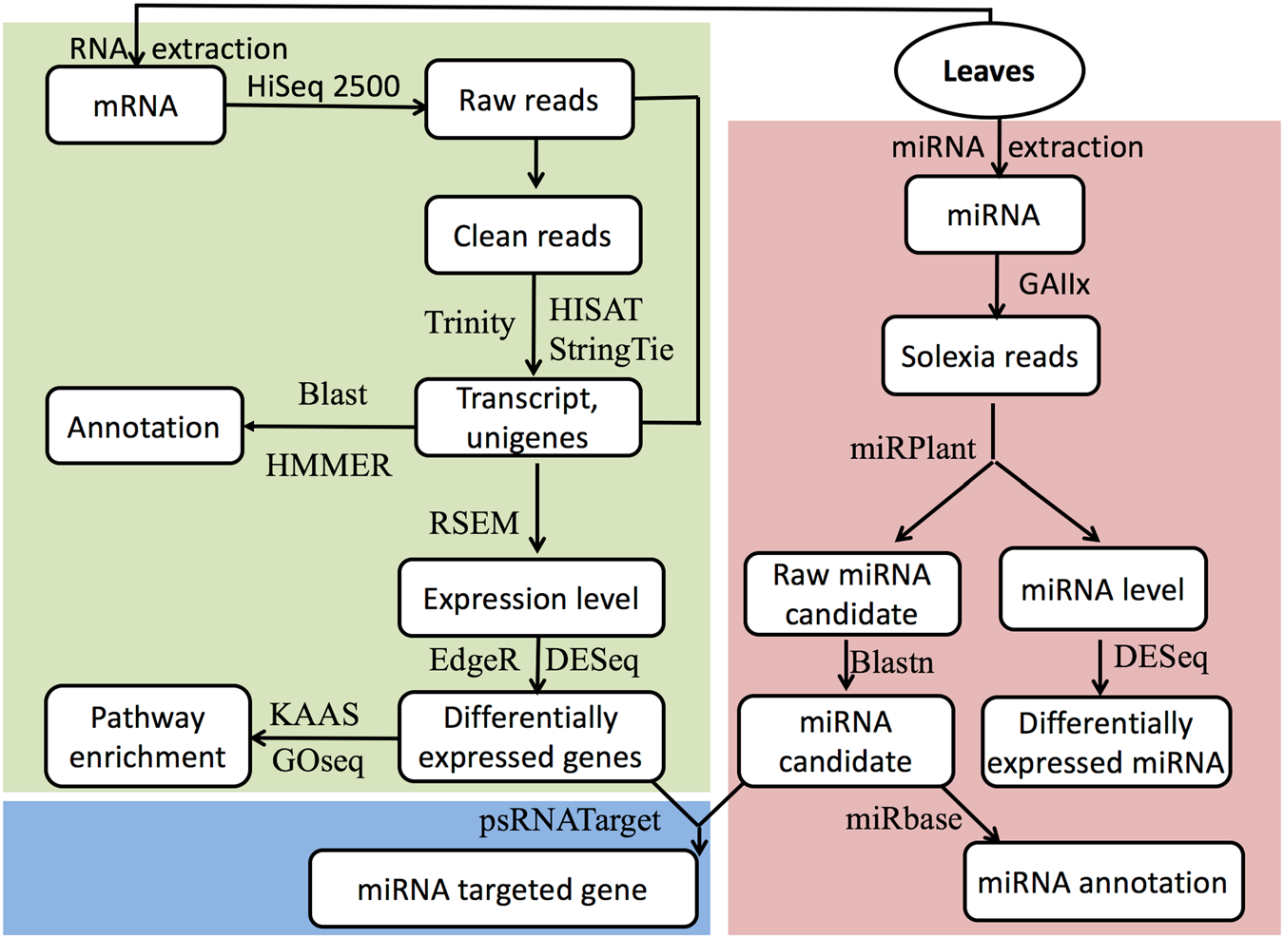
The workflow of mRNA and miRNA data analysis. The top left block in grass green shows the workflow of mRNA analysis. The bottom left block shows the workflow of prediction on miRNA and its mRNA target. The right block shows the workflow of miRNA analysis. The tools or software is given next to the line or pointer.

Pair-end PE-100-bp raw reads generated from Illumina HiSeq 2500 sequencing were preprocessed to remove adaptor sequences, filter out reads with >5% unknown bases, and remove low-quality reads (>20% of the bases with a quality score of less than 10). The retained reads, called clean reads, from the same experimental group were combined and assembled de novo to construct transcripts with Trinity software (version Trinityrnaseq_r20160317, https://github.com/trinityrnaseq) followed described protocols^42^. Clean reads were mapped back to transcript assembly, and poorly supported transcripts were removed as described previously^42^. Unigenes were defined as the longest sequence in an assembly cluster^42^. RSEM^76^ (version 1.2.27, https://deweylab.github.io/RSEM/) was adopted to quantify the total expressed transcripts in FPKM (Fragments per Kilobase of exon model per Million mapped reads) and raw counts. The R package EdgeR built in Trinity package^42^ was used to identify the differentially expressed mRNAs at least a 2-fold change and FDR value < 0.05. We also conducted a reference based assembling for the same batch of clean RNA-seq data by using HiSAT and StringTie followed published protocols^41^. The newest *N. tabacum* assembly V.4.5 was used as reference^40^. R package DEseq2^43^ (version 1.16, https://bioconductor.org/packages/release/bioc/html/DESeq2.html) was used to identify differentially expressed reference guided transcript and miRNA that exhibited at least a 2-fold change and P value < 0.05. Unigenes were then searched by Blatsn with an E-value threshold of E-5 against NR, SwissProt, GO, COG, KEGG and KOG databases and against Pfam database by HMMER with E-value of E-10. Online tool KAAS^44^ from KEGG (http://www.genome.jp/) was used to map expressed gene to K number and then convert to KO pathway number ID, called ko number, at database KEGG. The R package GOseq^45^ (http://bioconductor.org/packages/release/bioc/html/goseq.html) was used to conduct enrichment analysis on genes or miRNA the KEGG pathways at level P < 0.05. A schematic workflow of the analysis was presented (Fig. 6).

### microRNA analysis

microRNAs were extracted with a mirVana™ miRNA Isolation Kit (Ambion, Austin, TX, USA) and then used for library preparation with the Small RNA Sample Prep Kit (Illumina, SanDiego, CA, USA). miRNAs were sequenced by using the Solexa platform. Raw reads were first cleaned by using a corresponding Illumina process. Then, the cleaned reads were input into the software miRPlant^77^ (version 4, https://sourceforge.net/projects/mirplant/) to find miRNAs. The parameters were set to miR length 18–25 bp and minimum read count 4; other parameters were set to default. The adaptor sequence was 5′-AGATCGGAAGAGCACACGTCT-3′, and the reference genome sequence of *N. tabacum* K326 was downloaded from NCBI (version http://www.ncbi.nlm.nih.gov/nuccore/AWOJ00000000.1/)^78^. Potential non-miRNAs were removed if they were identified in the SiRNA, tRNA, snoRNA and rRNA database (pfam version 11, http://pfam.xfam.org/) after a Blastn searches with the following settings: E-value 10-5, -W 4 -F -G 2 -q -4. The remaining candidates were considered as miRNA. To annotate and compare conserved or reported miRNAs, the existing miRNAs in the most recent database miRbase (version 21.0, www.mirbase.org) were downloaded and used to identify the conserved or reported tabacum miRNAs by using the Blastn tool with a maximum of two mismatches allowed. A miRNA family code was then assigned to the mined miRNA if similarity was found according to described criteria^79^ and was then manually confirmed. A schematic workflow of the analysis is presented in Fig. 6.

### miRNA target analysis

Each miRNA target was predicted by using the tool psRNATarget^80^ (http://plantgrn.noble.org/psRNATarget/) with a strict parameter maximum expectation of 2 and other default settings as follows: length for complementarity scoring (hspsize) 20; target accessibility (UPE) 25.0; flanking length around the target site for target accessibility analysis 17; range of central mismatch leading to translational inhibition 9–11 nt.

### RT-qPCR analysis

Total RNA or miRNA was extracted from control or AHO-treated and pooled leaf samples of three to five plants used in RNA sequencing, respectively. There were at least three RT-qPCR reactions conducted for each sample. For mRNA expression analysis, the cDNA was generated using the HiScript II 1st Strand cDNA Synthesis Kit using random hexa-primers (Vazyme, China). For miRNA expression analysis, the stem-loop reverse transcription primers were designed as previously described^81,82^, and the reverse transcription reactions were performed as described for miRNA. qRT-PCR reactions were performed using FastStart Universal SYBR Green Master (Roche, China) on a BIOER FQD-96A instrument. The tobacco actin gene was used as reference gene for the normalization of reactions in the mRNA analysis^83^, and a tobacco U6 sequence was used as the reference for data normalization in the miRNA analysis^36^. The relative expression of each sample was calculated using the 2-ΔΔCT method^84^. Primers used in these qRT-PCR analyses are presented in Supplementary Table S6.

## Electronic supplementary material

Dataset 1

Dataset 2

Dataset 3
