# Supplementary material for: Integrating transcriptome and microRNA analysis identifies genes and microRNAs for AHO-induced systemic acquired resistance in N. tabacum
Source: Sci Rep. 2017 Oct 2;7:12504. doi: 10.1038/s41598-017-12249-y (PMC5624873; doi:10.1038/s41598-017-12249-y)
Supplement: Supplementary file 1 — Dataset 1 [file 41598_2017_12249_MOESM1_ESM.pdf]

Integrating transcriptome and microRNA analysis identifies genes and microRNAs for AHO-induced systemic acquired resistance in *N. tabacum*

Yongdui Chen[1#], Jiahong Dong[1#], Jeffrey L. Bennetzen[2,5], Micai Zhong[2], Jun Yang[3], Jie Zhang[1], Shunlin Li [4], Xiaojiang Hao[4], Zhongkai Zhang[1]*, Xuewen Wang[2,5]*

Supplementary information:

Table of Contents

I. Supplementary tables 2
II. Supplementary software, script and parameters 6

I. Supplementary tables

Table S1. Inhibitory effect of AHO on tomato spotted wilt virus in *N. tabacum* leaves

Treatment	Individual	Control		AHO		AHO		AHO		AHO	
AHO concentration (μg/ml)		0.00		1.25		2.50		5.00		10.00	
Numbers of necrotic lesions	1	109	146	64	63	25	12	22	20	18	15
	2	94	116	51	62	42	36	21	22	7	7
	3	121	140	25	33	18	24	7	6	24	16
	4	153	180	100	94	45	35	27	27	7	5
	5	136	160	85	52	40	26	18	23	15	20
Mean		271.0		125.8		60.6		38.2		26.8	
Standard error		20.6		21.8		9.0		6.8		5.8	
P<0.01		a		b		c		c		c	
Average inhibitory effect (%)		--		53.92		77.19		85.79		89.93	

TSWV was mechanically inoculated on two middle leaves of each plant 24 hours after spraying with AHO. The inhibitory effect was calculated as the percent of (the total necrotic lesions in each control plant – that in each AHO-treated plant) / the total number of necrotic lesions in each control plant×100%. One-way ANOVA was used for statistical analysis between treatment and control. Letter a, b, and c represent significance compared to each other at level 0.01.

Table S2. Summary of RNA-seq reads and assembly for *Nicotiana tabacum* K326

	Category	Data			
RNA-seq reads	Sample ID	Control_1	Control_2	AHO_1	AHO_2
	Reads	21,124,948	21,284,899	20,648,828	20,694,821
	Mapping percent	76.62%	76.52%	74.10%	73.92%
	GC(%)	42.95	42.95%	42.94%	42.95%
	Q30(%)	92.00%	91.80%	91.30%	91.10%
	Total reads		83,753,496		
		**De novo Trinity Assembly**		**Referenced guided assembly**	
Transcripts	Total number	236,647		99,547	
	count (>600 bp)	26,972		68,455	
	Mean length (bp)	1,081		1,255	
	N50 length (bp)	1,775		1,791	
Unigenes	Total number	95,422		66,700	

Data shows the statistical information of RNA-seq PE-100 reads from Illumina Hiseq 2500 sequencing and assembly by de novo method with from software Trinity or by reference guided with StringTie. The total number of clean reads is the sum of reads from each sample.

Table S3. Unigene annotation by searching against public databases

Database	Annotated_Number	300<=Length<1000	Percentage	Length>=1000	Percentage
COG	7,764	1,988	2.1%	5,135	5.4%
GO	15,543	5,422	5.7%	7,563	7.9%
KEGG	10,255	3,588	3.8%	5,135	5.4%
KOG	17,208	5,945	6.2%	8,571	9.0%
Pfam	19,623	6,047	6.3%	11,611	12.2%
Swissprot	18,941	6,628	6.9%	9,680	10.1%
nr	35,854	13,962	14.6%	14,334	15.0%
All_annotated	36,073	14,059	14.7%	14,347	15.0%
Total Unigenes	95,422				

The above table shows the numbers and percentage of unigenes annotated in databases. The number shown in the last row is the total count of annotated genes after removing duplicated unigenes.

Table S4. miRNA identification in N. tabacum

Excel file: STable4 miRNA_and_DEM.xls

Table S5. Predicted miRNA regulated target genes with differential expression

Excel file: Stable5 target.DEG.2fold_Vs_miRNAseq_k326_family_updated.xls

Table S6. **Primers for qRT-PCR validation of miRNA and mRNA expression**

ID	RT primer (5'-3')	Forward primer (5'-3')	Universal primer (5'-3')
**miRNAs**			
miR156v	GTCGTATCCAGTGCAGGGTCCGAGGTATTCGCACTGG ATACGACGTGCTC	GCG CGC GTT GAC AGA AGA TAG A	ATCCAGTGCAGGGTCCGAGG
miR172f	GTCGTATCCAGTGCAGGGTCCGAGGTATTCGCACTGG ATACGACATGCAG	GCG CGC GAG AAT CTT GAT GAT G	ATCCAGTGCAGGGTCCGAGG
miR172g	GTCGTATCCAGTGCAGGGTCCGAGGTATTCGCACTGG ATACGACATGCAG	GCG CGC GAG AAT CTT GAT GAT G	ATCCAGTGCAGGGTCCGAGG
miR7997	GTCGTATCCAGTGCAGGGTCCGAGGTATTCGCACTGG ATACGACCATTTT	GCG CGT TGC TCG GAC TCT TCA	ATCCAGTGCAGGGTCCGAGG
novel101	GTCGTATCCAGTGCAGGGTCCGAGGTATTCGCACTGG ATACGACTTTTTG	CGC GCG ATT CTT TTT TGA ACG GAC	ATCCAGTGCAGGGTCCGAGG
novel152	GTCGTATCCAGTGCAGGGTCCGAGGTATTCGCACTGG ATACGACGAGAGT	CGC GAA GGT CTG CGT ACA CAT T	ATCCAGTGCAGGGTCCGAGG
novel156	GTCGTATCCAGTGCAGGGTCCGAGGTATTCGCACTGG ATACGACGGGTAT	TGC GAG AGA GGC TGT TTC CGA T	ATCCAGTGCAGGGTCCGAGG
novel98	GTCGTATCCAGTGCAGGGTCCGAGGTATTCGCACTGG ATACGACAATATA	GCG CGT TGT TGG ATC CGT AGT AT	ATCCAGTGCAGGGTCCGAGG
U6		TTGGAACGATACAGAGAAGATTAGC	AATTTGGACCATTTCTCGATTTGTG
**Genes**			
c56968.g_c0		GCAAATGCCCACTCAGGTTG	GACGCATTTGTTGAGGGTGC
c58965.g_c0		GACCAAACAAGCACTCGCAA	CTGGAGGGATCATTGGTTTTGG
c64248.g_c1		TAGTGTGTACGCAGACCTTACCCT	GAGTTTGTTTTCTGGTTTCATGCGT
c43078.g_c0		GGAGAGGGTAGTGTGTTCGCAG	AAGCTCCTCCTACACGAGTCCT
c44105.g_c1		TCCGAGGAAGAAACTGAGTCGAGG	AATATAAGCGGGGTATGGGGAGG
c45235.g_c0		GGCAGTCCACAGAGAAAGGG	CACTAGTGGGACCTGGGAGG
c56252.g_c0		CCGGCATGTAATTCTGCTGGAATG	GACACCAAGAAGGCATAGTCGAGG
Actin		TTGGAACGATACAGAGAAGATTAGC	AATTTGGACCATTTCTCGATTTGTG

II. Supplementary software, script and parameters

- **De novo transcript assembly:**

  Software: Trinityrnaseq, version r20160317,

  Parameters (default otherwise described here): --min_contig_length 200, --group_pairs_distance 500

  Others default

  Script and instruction is described from published protocol [1] , web link

  http://www.nature.com/nprot/journal/v8/n8/full/nprot.2013.084.html

- **Reference guided transcript assembly:**

  Software Hisat (version 2.0.5) and StringTie (version 1.3.3b)

  Script and instruction is described from published protocol [2] , web link

  http://www.nature.com/nprot/journal/v11/n9/full/nprot.2016.095.html

- **RSEM (bundled in Trinity package): v5.10.1**

  Parameters: default

  Script and instruction is described from published protocol [1] , web link

  http://www.nature.com/nprot/journal/v8/n8/full/nprot.2013.084.html

- **Blast analysis:**

  Tool: BLAST, version 2.2.31, (default otherwise described here):

```
####this script is used to annotate unigene
# the unigeneSequence.fa. for the query sequence
p="unigeneSequence.fa"
#set up the downloaded database to variable $d such as Nr
d="Nr"
# sourcedir is for unigenes directory
#output format "6 std" and added column qlen for query length
blastn -query $p -db $d -evalue 1e-5 -outfmt '6 std qlen' -penalty -4 -gapopen 2 -gapextend 2 \
```

-best_hit_overhang 0.25 -word_size 10 -num_threads 8 -out $p-$d.blastout

####this script is to identify tRNA, snoRNA and rRNA database pfam .
#$q is the file name of the raw miRNA canidates' sequence
#pfam is the database
#results saved in file pfam_similarRNA.blastout in plain text
Blastall -p blastn –F -i $q –d pfam -e 1e-5 -W 4 -G 2 -q -4 -m 8 -o pfam_similarRNA.blastout

Blast2go: version 2.5, (default otherwise described here): E-value 1e-5

- **Differentially expressed gene or miRNA:**
  1. EdgeR (bundled in Trinity package): FC 2, FDR 0.05
  Script and instruction is described from published protocol [1] , web link
  http://www.nature.com/nprot/journal/v8/n8/full/nprot.2013.084.html

  2. DEseq, version 1.16: Fold change 2, P 0.05

     1) script Name: DEseq2_script.R

```
#usage: copy the following lines and paste into R console
## For DEseq2 differential expression analysis of miRNA and mRNA in this study
## For differential expression graph plot
#set working dir
setwd('E:/DEseq/script')
#get read counts called cts, and sample information called coldata
cts <- read.csv(file="miRNA_count.csv", header=TRUE)
rownames(cts)<- cts[,1]
cts<- cts[,-1]
head(cts,3)
##              Control   AHO
```

```
##novelMiR_11821   44899     0
##novelMiR_11548   44899     0
##novelMiR_11950   39784 37664
coldata <- read.table(file="colData.txt", header=TRUE)
head(coldata,5)
##        group treatment
## 1 Tab_Control_S1   Control
## 2     Tab_AHO_S2       AHO
library("DESeq2")
coldata$treatment = factor(x = coldata$treatment,levels = c('Control', 'AHO'))
#construct DESeq object
dds = DESeqDataSetFromMatrix(countData = cts, colData = coldata, design = ~ treatment)
dds = DESeq(dds)
res = results(dds)
write.table(res, file="DEM_Deseq_result.txt", sep="\t",col.names=NA)
##filtered results of differential expression FC >=2, P<=0.05
DEM_FC2P0.05 <- subset(res, abs(log2FoldChange)>=1 & pvalue<=0.05)
write.table(DEM_FC2P0.05, file="DEM_Deseq_result_FC2P0.05.txt", sep="\t",col.names=NA)
```

2) script Name: Vocanal_plot.R

```
##plot graph
##construct data frame with fold change and p value from res or input data file
tab = data.frame(log2FC = res$log2FoldChange, negLogPval = -log10(res$pvalue))
par(mar = c(5, 4, 4, 4))
plot(tab, pch = 16, cex = 0.6, xlab = expression(log[2]~FC), ylab = expression(-log[10]~pvalue))
signGenes = (tab$log2FC > 1 & tab$negLogPval > -log10(0.05))
points(tab[signGenes, ], pch = 16, cex = 0.5, col = "red")
signGenes = (tab$log2FC < -1 & tab$negLogPval > -log10(0.05))
points(tab[signGenes, ], pch = 16, cex = 0.5, col = "green")
```

```
abline(h = -log10(0.05), col = "orange", lty = 2)
abline(v = c(-1, 1), col = "blue", lty = 2)
```

3) script Name: GOseq_script.R

```
library(goseq)
##all DEseq output as input here
all.genes <- read.table(file="DEG_Deseq_result.gene.txt", header=TRUE)
all.genes <- as.data.frame(all.genes)
rownames(all.genes) <- all.genes[,1]
genes <- as.integer(all.genes$padj < 0.05 & abs(all.genes$log2FoldChange)>=1)
names(genes) <- row.names(all.genes)
genes <- na.omit(genes)
glength<- read.table(file="DEG_Deseq_result.gene.filter.id.list.len", header=FALSE)
glen <- glength[,2]
pwf = nullp(genes, bias.data=as.vector(glen))
getko <- read.table("DEG_Deseq_result.gene.filter.id.list.go.NA.txt", header=F, sep="\t", fill=T)
GO.wall <- goseq(pwf, gene2cat=getko[,c(1,2)])
##> head(GO.wall,3)
##     category over_represented_pvalue under_represented_pvalue numDEInCat numInCat
##    category over_represented_pvalue under_represented_pvalue numDEInCat numInCat
##67   ko04111            0.001520392                0.9997066        22       26
##8    ko00196            0.003058524                0.9989970        28       39
##68   ko04113            0.009473834                0.9985285        14       16
```

4) script Name: heatmap_script.R

```
##construct a log2FPKM data file
##instruction from https://www.rdocumentation.org/packages/gplots/versions/3.0.1/topics/heatmap.2
```

```
#### the R script to make the heatmap
## to prepare data for heatmap
##to run this part within R console
data <-read.table("degFPKMPlus1log2.txt", header=TRUE, sep="\t")
mat_data <- data.matrix(data[,2:ncol(data)])
rownames(mat_data) <- data[,1]
my_palette <- colorRampPalette(c("green", "yellow", "red"))(n = 60)
heatmap.2(mat_data,
  labRow = "",
  key.xlab ="",
  keysize = 1,
  density.info="none",
  trace="none
  scale="col",
  margins =c(9,9),
  col=my_palette,
  dendrogram="both",
  key= TRUE,
  key.par=list(mar=c(4.5,0.4,1, 0.2)),
  )
```

References

1 Haas, B. J. *et al.* De novo transcript sequence reconstruction from RNA-seq using the Trinity platform for reference generation and analysis. *Nature protocols* **8**, 1494-1512, doi:10.1038/nprot.2013.084 (2013).

2 Pertea, M., Kim, D., Pertea, G. M., Leek, J. T. & Salzberg, S. L. Transcript-level expression analysis of RNA-seq experiments with HISAT, StringTie and Ballgown. *Nat. Protocols* **11**, 1650-1667, doi:10.1038/nprot.2016.095 (2016).
